# A Pattern of Early Radiation-Induced Inflammatory Cytokine Expression Is Associated with Lung Toxicity in Patients with Non-Small Cell Lung Cancer

**DOI:** 10.1371/journal.pone.0109560

**Published:** 2014-10-07

**Authors:** Shankar Siva, Michael MacManus, Tomas Kron, Nickala Best, Jai Smith, Pavel Lobachevsky, David Ball, Olga Martin

**Affiliations:** 1 Division of Radiation Oncology and Cancer Imaging, Peter MacCallum Cancer Centre, Melbourne, VIC, Australia; 2 Sir Peter MacCallum Department of Oncology, the University of Melbourne, Melbourne, VIC, Australia; 3 Physical Sciences, Peter MacCallum Cancer Centre, Melbourne, VIC, Australia; 4 Molecular Radiation Biology Laboratory, Peter MacCallum Cancer Centre, VIC, Australia; University of Pittsburgh School of Medicine, United States of America

## Abstract

**Purpose:**

Lung inflammation leading to pulmonary toxicity after radiotherapy (RT) can occur in patients with non-small cell lung cancer (NSCLC). We investigated the kinetics of RT induced plasma inflammatory cytokines in these patients in order to identify clinical predictors of toxicity.

**Experimental Design:**

In 12 NSCLC patients, RT to 60 Gy (30 fractions over 6 weeks) was delivered; 6 received concurrent chemoradiation (chemoRT) and 6 received RT alone. Blood samples were taken before therapy, at 1 and 24 hours after delivery of the 1^st^ fraction, 4 weeks into RT, and 12 weeks after completion of treatment, for analysis of a panel of 22 plasma cytokines. The severity of respiratory toxicities were recorded using common terminology criteria for adverse events (CTCAE) v4.0.

**Results:**

Twelve cytokines were detected in response to RT, of which ten demonstrated significant temporal changes in plasma concentration. For Eotaxin, IL-33, IL-6, MDC, MIP-1α and VEGF, plasma concentrations were dependent upon treatment group (chemoRT vs RT alone, all *p-*values <0.05), whilst concentrations of MCP-1, IP-10, MCP-3, MIP-1β, TIMP-1 and TNF-α were not. Mean lung radiation dose correlated with a reduction at 1 hour in plasma levels of IP-10 (*r^2^ = *0.858, *p*<0.01), MCP-1 (*r^2^* = 0.653, *p*<0.01), MCP-3 (*r^2^* = 0.721, *p*<0.01), and IL-6 (*r^2^* = 0.531, *p* = 0.02). Patients who sustained pulmonary toxicity demonstrated significantly different levels of IP-10 and MCP-1 at 1 hour, and Eotaxin, IL-6 and TIMP-1 concentration at 24 hours (all *p-values* <0.05) when compared to patients without respiratory toxicity.

**Conclusions:**

Inflammatory cytokines were induced in NSCLC patients during and after RT. Early changes in levels of IP-10, MCP-1, Eotaxin, IL-6 and TIMP-1 were associated with higher grade toxicity. Measurement of cytokine concentrations during RT could help predict lung toxicity and lead to new therapeutic strategies.

## Introduction

Lung cancer is the leading cause of cancer-related death in both sexes [1]. Non-small cell lung cancer (NSCLC) accounts for 85% of cases. Radiotherapy (RT), alone or in combination with chemotherapy, is a standard definitive treatment approach for patients with locally advanced NSCLC or inoperable patients with early stage disease [2], [3]. Over half of NSCLC patients are currently treated with RT. This rate may increase in the future with the optimal RT utilization rate being estimated to be 76% [4]. However, local failures are a major cause for the relatively poor survival reported for patients treated with RT. A recent meta-analysis suggests that local failures still occur in up to 38% of patients [5]. Efforts to intensify RT, however, are severely limited by the need to constrain dose to the surrounding normal lung in order to preserve function [6]. Lung toxicity caused by RT (termed *pneumonitis*) is a real and potentially debilitating toxicity, sometimes leading to patient death [7]. In the modern era symptomatic pneumonitis still occurs in 29.8% of patients and fatal pneumonitis in 1.9% [8]. Currently used RT planning constraints that were designed to limit the risk of pneumonitis are based on evidence over a decade old [9]. These constraints apply to populations and give no indication of an individual patient’s susceptibility to lethal toxicity, beyond the fact that on average higher RT doses to larger volumes are more likely to be toxic. It is therefore imperative to establish *in vivo* biomarkers for prediction or early assessment of pneumonitis that will ultimately assist in avoiding RT induced lung dysfunction by individualizing treatment.

The pathophysiology of radiation-induced lung toxicity is incompletely understood at present. A large body of evidence from animal models, molecular biology and clinical observations suggests that normal tissue injury is a dynamic and progressive process [10], [11]. A complex interaction between radiation-induced damage to parenchymal cells, supporting vasculature and associated fibrotic reactions results in acute and late radiation toxicities. In the lung, these changes can manifest themselves as reduced pulmonary function and in a chronic inflammatory cascade known as pneumonitis [12]. There are many factors that influence the likelihood of severe respiratory toxicity including the volume of irradiated parenchyma, pre-existing lung disease and the use of radiosensitizing chemotherapy [13]. However, the exact biological mechanisms of inflammatory cascade and eventual pulmonary fibrosis are not fully elucidated.

Cytokine release in response to ionizing radiation is a documented phenomenon and may play a major role in subsequent radiation induced lung toxicity (reviewed in [14]–[18]. A non-specific acute reaction, or “cytokine storm” usually resolves within 24 hours [19]. Fractionated radiation, however, creates a constant complex stress response and a cytokine profile is different to that induced by a single radiation dose [20]. RT-related plasma concentrations of one or more cytokines in humans have correlated with lung toxicity. Transforming growth factor (TGF)-β1 [21]–[24], interleukin (IL)-6 and IL-10 [25], [26] during RT have been suggested as possible risk markers in these studies. However, other studies have reported contradictory or negative findings [27], [28].

The rationale for the composition of our panel of 22 potential biomarkers for lung tissue toxicity was based on several published reports dissecting inflammatory and radiation response. The plasma levels of a range of cytokines have been previously investigated in context of both murine [29] and cell models [20]. A range of pro-inflammatory cytokines are expressed as acute phase reactants, including tumour necrosis factor (TNF)-α, i IL-1 and IL-6 [14], [18]. Chemokines act as chemoattractants for leukocytes which potentiate the inflammatory response, such as interferon-inducible protein-10 (IP-10) which attracts predominantly neutrophils, macrophage inflammatory protein (MIP)-1α, and macrophage chemoattractant protein (MCP)-3 which attracts predominantly monocytes, and MIP-1β and MIP-3α which attract predominantly lymphocytes [16], [17]. Induction of MIP-3β results in chemoattraction of dendritic cells and antigen engaged B-cells [30]. MCP-1 is a cytokine that has been associated with many inflammation-related diseases and has been implicated in the progression and prognosis of several cancers [29], [31]. Upregulation of MCP-3 gene expression has been shown to be maximal at 1-hour in response to radiation in rat liver [32]. Excessive release of interferon-gamma (IFNγ) has been associated with the pathogenesis of chronic inflammatory and autoimmune diseases [17]. Macrophage-derived chemokine (MDC), is involved in chronic inflammation and dendritic cell and lymphocyte homing [17]. Eotaxin is a chemoattractant for eosinophils and is implicated in acute inflammatory lung injury responses, particularly in emphysema and asthma [33], [34]. IL-3, IL-11, IL-22 and IL-33 are all acute phase reactants that potentiate cellular immune signalling and inflammatory responses [35]–[37]. The induction of all these inflammatory cytokines in response to radiation stimulate the subsequent expression of fibrotic cytokines such as the TGF-β family and vascular endothelial growth factor (VEGF). These in turn facilitate the progression from pneumonitis to lung fibrosis [38], [39]. Helping to balance this process, both IL-22 and IL-10 can act to down-regulate the pneumonitic response by blocking pro-inflammatory cytokines and function of antigen-presenting cells [25], [37]. Additionally, tissue inhibitors of metalloproteinase (TIMP)-1 acts to down-regulate the profibrotic response and is elevated in chronic inflammatory disease states [29], [40].

In this study, we report the modulation of plasma concentrations of these cytokines in patients receiving RT alone or RT with concurrent radiosensitising chemotherapy. In contrast to many previous studies, we consider the differential patterns of response in patients receiving radiosensitizing chemotherapy compared to those receiving RT alone. We assess a homogenous cohort of patients receiving identical dose/fractionation schedules, and employ a large panel of candidate cytokines. Additionally, we report the effect of treatment volume and dose to normal lung tissue on plasma cytokine concentrations, suggesting that these cytokines could be used as *in-vivo* ‘biodosimeters’ of individual radiation dose. Finally, we identified five cytokines that that could be predictive of pulmonary lung toxicity and should be validated in a larger cohort as early predictive markers for clinical radiation pneumonitis.

## Materials and Methods

This research was the translational component of an institutional ethics committee approved prospective clinical trial at the Peter MacCallum Cancer Centre (Universal Trials Number U1111-1138-4421). All patients provided written consent to participate in this study. Consecutive patients undergoing definitive RT with or without concurrent chemotherapy underwent serial venipuncture and blood collection for inflammatory cytokine testing. Patients were followed up at three monthly intervals after treatment. Toxicity scoring was performed prospectively at each clinical visit using Common Terminology Criteria for Adverse Events (CTCAE) version 4.0.

### Radiotherapy

All patients were planned to receive 60 Gy in 30 fractions of RT delivered over 6 weeks using 3D conformal techniques. Respiratory-sorted four-dimensional computed tomography (4DCT) was used for RT planning. Target delineation was performed on an Elekta FocalSim workstation (Stockholm, Sweden). An internal target volume (ITV) was delineated from the maximal intensity projection (MIP) series, and a further isotropic expansion of 5 mm expansion was used to generate the clinical target volume, and a further 10 mm isotropic expansion was used to create the planning target volume (PTV). The lung organ at risk volume was defined as the volume of both lungs minus the volume of the ITV. Typically a 3–4 field RT technique using 6MV photons was used with effort made to avoid the contralateral unaffected lung and spare spinal cord whilst ensuring the PTV was within −5% and +7% of the prescribed dose, as per ICRU 62 recommendations. Dose constraints to organ at risks dose were as follows: spinal canal ≤45 Gy, mean lung dose ≤20 Gy, the volume of lung receiving 5 Gy (V5) ≤60%, V20≤35%, V30≤30%. In patients receiving concurrent chemotherapy, this was delivered using platinum doublets. This consisted of either 2×3 weekly cycles of 50 mg/m^2^ cisplatin days 1 and 8, with 50 mg/m^2^ etoposide days 1–5, or 6x weekly cycles of carboplatin AUC 2 day 1 with 45 mg/m^2^ paclitaxel day 1. The first cycle of concurrent chemotherapy was commenced immediately prior to the first fraction of radiotherapy. No patient received adjuvant chemotherapy after the concurrent chemotherapy delivery. All patients in our institution are planned for concurrent chemotherapy unless precluded by cardiovascular comorbidities or renal insufficiency.

### Blood Sample Processing

Patient blood samples were collected and processed at five time points in this study. Baseline blood samples were collected before therapy, and 4 consecutive samples were collected at 1 hour after the first fraction of RT, 24 hours after the first fraction of RT, 4 weeks into the course of RT, and 12 weeks after the completion of RT. The early time points were chosen for pragmatic purposes to reflect clinical practicality; patients are routinely within the department within 1 hour of the first and second fraction of RT. These times allow for the possibility of early adaptation of the RT plan based on cytokine response. The 4 week time point was chosen as this typically coincides with approximately 40 Gy of delivered dose, which allows an ideal opportunity for adaptive radiation planning prior to completion of RT [41]. The final time point, at 12 weeks after completion of RT, coincides with the period in which fibrosing alveolitis and the clinical manifestation of subsequent pneumonitis can occur [42]. Blood was collected in 9 mL ethylenediaminetetraacetic acid (EDTA) tubes, and centrifuged twice at 2000 rpm for 10 minutes and then at 4000 rpm for 10 minutes. The upper 90% of the plasma was transferred into 2 mL aliquots into cryovials and stored at –80°C. These were subsequently processed in batch using a commercial flow cytometry system.

### Cytokine Analysis

Each patient sample was run in duplicate using 100 µl of plasma diluted by a factor of 2. A commercial multiplexed sandwich ELSIA-based array was used (Quantibody custom array, RayBiotech Inc., Norcross, GA, USA). All of the samples were tested using a panel of 22 cytokines: Eotaxin, IFNγ, IL-6, IL-10, IL-11, IL-22, IL-3, IL-33, IP-10, MCP-1, MCP-3, MDC, MIP-1α, MIP-1β, MIP-3α, MIP-3β, TGF-β1, TGF-β2, TGF-β3, TIMP-1, TNF-α, VEGF. The antibody array is a glass-chip-based multiplexed sandwich ELISA system designed to determine the concentrations all 22 cytokines simultaneously. One standard glass slide was spotted with 16 wells of identical biomarker antibody arrays. Each antibody, together with the positive and negative control, was arrayed in quadruplicate. The samples and standards were added to the wells of the chip array and incubated for 3 h at 4 °C. This was followed by three to four washing steps and the addition of primary antibody and HRP-conjugated streptavidin to the wells. The signals (Cy3 wavelengths: 555 nm excitation, 655 nm emission) were scanned and extracted with a Genepix laser scanner (Axon Instruments, Foster City, CA), and quantified using Quantibody Analyzer software (Ray Biotech Inc). Each signal was identified by its spot location. The scanner software calculated background signals automatically. Concentration levels, expressed in picograms per milliliter (pg/ml), were calculated against a standard curve set for each biomarker from the positive and negative controls.

### Statistical Methods

Patients were grouped into those receiving concurrent chemotherapy (chemoRT) and those receiving RT alone. Two-way ANOVA assuming repeated measures testing for different time points was used to assess differences in cytokine concentrations between chemoRT and RT groups and across sampled time points. These changes from baseline cytokine concentration were measured at an individual patient level. Subsequently, 95% confidence intervals were calculated and corrections for multiple comparisons were performed using Dunnett’s method and an alpha of 0.05. Clinical toxicities secondary to treatment were assessed using CTCAE v4.0 at baseline, 4 weeks into treatment and 12 weeks after treatment completion. The PTV and mean lung dose (MLD) of RT were recorded for each patient and correlated with the change in cytokine concentrations from baseline at one hour after the first fraction, four weeks into treatment and 12 weeks after treatment completion using a linear regression model. Patients were dichotomized into those experiencing severe respiratory toxicity (grade 2+) and those who did not. Unpaired two-tailed *t-tests* were used to compare the mean cytokine concentrations at these timepoints between the patient toxicity groups. All statistical analyses were performed using PRISM v6.0 software.

## Results

Twelve patients were enrolled into this study with a median age of 67 years (range 46–89 years). All patients received 60 Gy in 30 fractions of RT. Six patients received concurrent chemotherapy and six received RT alone (due to comorbidities). Six patients had stage III disease, three had stage II disease and three had stage I disease. Patient population characteristics are listed in **Table 1**. Individual patient characteristics are further given in **Table S1**.

**Table 1:** Patient Characteristics.

Characteristic	Number (%)
Sex	
Male	8 (67%)
Female	4 (33%)
Age	
Median	67 years
Range	46–89 years
Planning Target Volume (PTV)	
Median	320 cm^3^
Range	87 cm^3^–1138 cm^3^
Mean Dose to Normal Lung	
Median	11.7 Gy
Range	6.0 Gy–19.1 Gy
Total Radiation Dose	60 Gy (100%)
Clinical Stage	
I	3 (25%)
II	3 (25%)
III	6 (50%)
Histology	
Squamous Cell Carcinoma	6 (50%)
Adenocarcinoma	3 (25%)
Non-Small Cell [NOS*]	2 (17%)
Large Cell Neuroendocrine	1 (8%)
Treatment:	
RT alone	6 (50%)
Concurrent Cisplatinum/Etoposide	1 (8%)
Concurrent Carboplatinum/Paclitaxel	5 (42%)

*NOS – Not Otherwise Specified.

### Effect of Treatment Group and Sample Time Point

**Figure 1:**
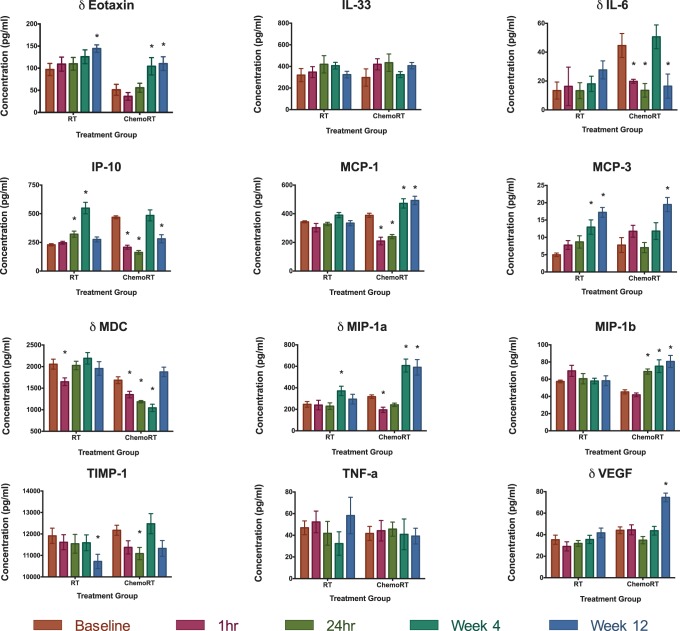
Mean plasma (+/− standard error) cytokine levels at each time point during radiotherapy (1-hour, 24-hours, 4-weeks) and after radiotherapy (12-weeks). Levels are grouped into treatment type (ChemoRT vs RT alone). Cytokines in which the variation is different dependent upon treatment groups are marked with a *delta* (*δ*). Within each cytokine graph, an *asterisk* (*) denotes at which time the level of plasma cytokines are significantly different from the baseline level, with corrections for multiple comparisons performed using Dunnett’s method.

**Figure 2:**
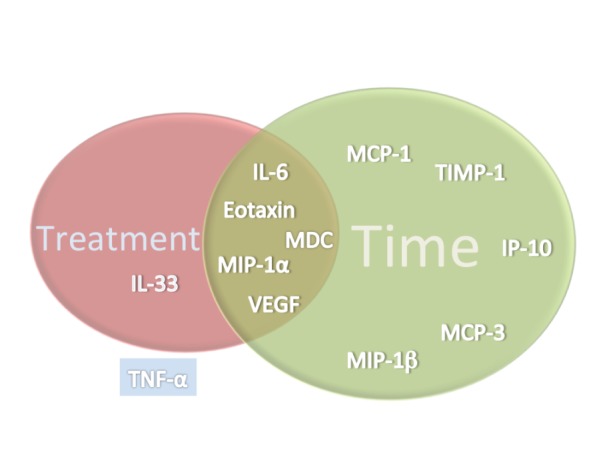
Venn diagram depicting the relationship between variation of plasma cytokine levels and the variables of time and treatment. MCP-1, TIMP-1, IP-10, MCP-3 and MIP-1β vary differently across the different timepoints sampled, but are have similar variance between treatment groups (RT alone vs ChemoRT). IL-6, Eotaxin, MDC, MIP-1α and VEGF levels varied across both time and treatment group. TNF-α levels were no different across time points sampled or treatment delivered.

Of the 22 cytokines analysed, results from 12 cytokines were above the limit of detection. These were Eotaxin, IL-33, IL-6, MCP-1, MDC, MIP-1α, VEGF, IP-10, MCP-3, MIP-1β, TIMP-1 and TNF-α. Of these 12 cytokines, all except for IL-33 and TNF-α demonstrated significant variation in concentrations across the different time points (all *p*-*values* ≤0.02, **Table S2**). The absolute changes in concentrations for each of the 12 plasma cytokines are depicted in **Figure 1** **.** Levels of Eotaxin, IL-33, IL-6, MDC, MIP-1α and VEGF were different in those patients receiving chemoRT as compared to those receiving RT alone (all *p-values* <0.01), whilst concentrations of IP-10, MCP-1, MCP-3, MIP-1β, TIMP-1 and TNF-α were not dependent upon the treatment group. In the RT alone group, the peak change in cytokine levels (depression or elevation) was seen at 4 weeks during treatment for IL-33, IP-10, MCP-1, & MIP-1α. The peak change in cytokine level was seen at 12 weeks after treatment completion for Eotaxin, IL-6, MCP-3, TIMP-1 and VEGF. By comparison, in the chemoRT group, the peak change in cytokine levels (depression or elevation) was seen at 4 weeks during treatment for MDC and MIP-1α only. The peak change in cytokine level was seen at 12 weeks after treatment completion for Eotaxin, MCP-3, MIP-1β, and VEGF. There was significant interaction between treatment group and sample time point (*p-values* <0.01) for concentrations of IL-6, IP-10, MCP-1, MDC, MIP-1α, MIP-1β and VEGF, indicating that the variation of plasma cytokine concentrations over time was not the same for the RT group as for the chemoRT group. Conversely, there was no significant interaction between treatment groups and sample time points for Eotaxin, MCP-3 and TIMP-1 indicating that the variation of cytokine concentrations across time was the same for both treatment groups. A summary of the cytokine levels that varied by time and those that varied by treatment group are depicted in **Figure 2**.

### Effect of Mean Lung Dose and PTV volume

**Figure 3:**
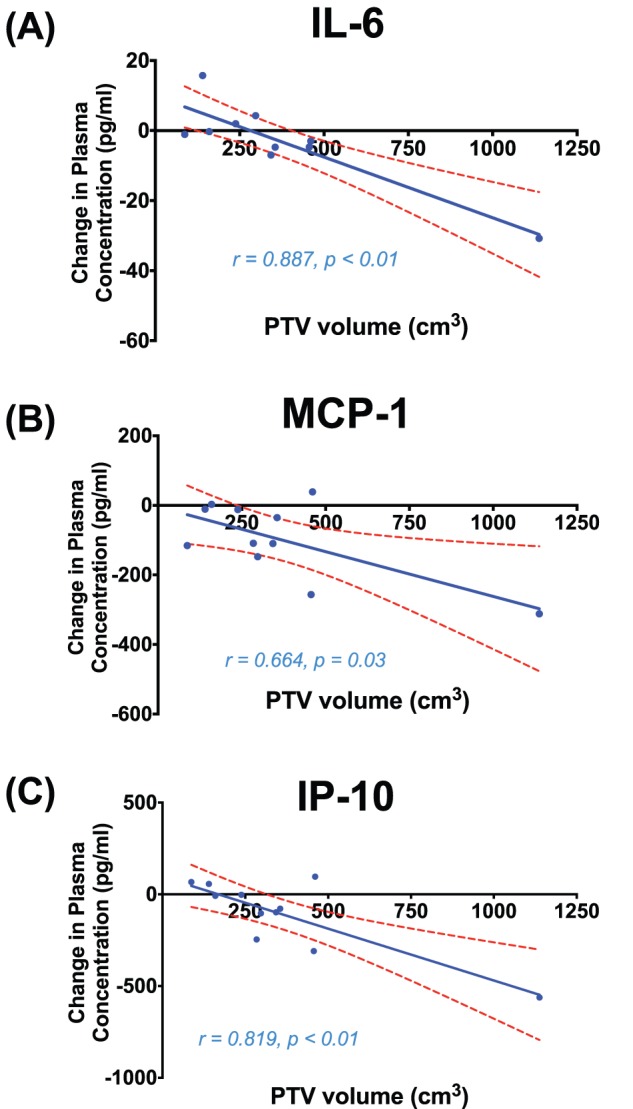
Correlation between irradiated PTV volume and the change in plasma cytokine concentration from baseline to 1-hour post irradiation for IL-6 (A), MCP-1 (B), and IP-10 (C). Linear-regression line (blue) is displayed with 95% confidence intervals (red broken line).

The median (range) PTV volume in all patients was 320 cm^3^ (87 cm^3^–1138 cm^3^). Plasma concentrations decreased from baseline at 1-hour post irradiation in all patients in a linear volume dependent manner for IL-6 (*r* = 0.887, *p<*0.01), MCP-1 (*r* = 0.664, *p* = 0.03), and IP-10 (*r* = 0.819, *p*<0.01), which is depicted in **Figure 3**. The extent of the reduction in these plasma cytokine concentrations correlated with irradiated target volume. The strongest correlation was observed for IL-6 (**Figure 3A**). Change in plasma concentration at 1-hour in the 9 remaining cytokines did not correlate with PTV volume. The changes in plasma concentration from baseline for all 12 cytokines did not correlate with irradiated target volume at either 4 weeks into treatment or 12 weeks after treatment.

**Figure 4:**
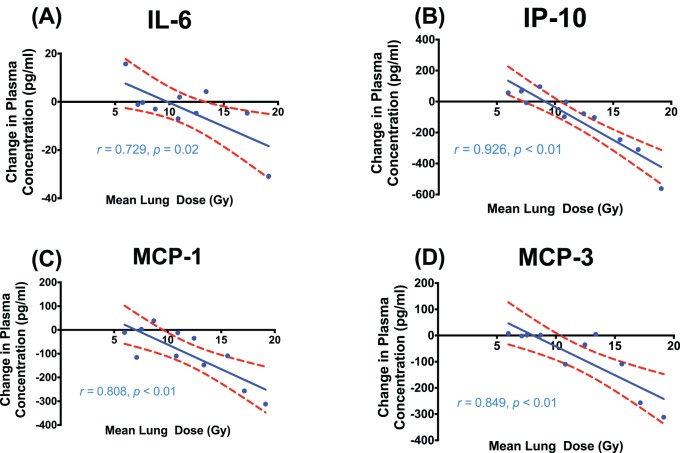
Correlation between mean lung dose (MLD) and the change in plasma cytokine concentration from baseline to 1-hour post irradiation for IL-6 (A), IP-10 (B), MCP-1 (C) and MCP-3 (D). Linear-regression line (blue) is displayed with 95% confidence intervals (red broken line).

The median (range) MLD in all patients was 11.7 Gy (5.97 Gy–19.14 Gy). Similar to the effect seen with PTV volume, plasma concentrations decreased from baseline at 1-hour post irradiation in a linear dose dependent manner for IL-6 (*r = *0.729, *p* = 0.02), MCP-1 (*r = *0.808, *p*<0.01), and IP-10 (*r = *0.926, *p*<0.01), depicted in **Figure 4**. In addition, MCP-3 also demonstrated linear reduction in plasma concentration at 1-hour for a given MLD (*r = *0.849, *p*<0.01). The MLD was proportional to a reduction in these plasma cytokine concentrations at 1-hour. Change in plasma concentration at 1-hour in the 8 remaining cytokines did not correlate with MLD. Again, the change in plasma concentration from baseline for all 12 cytokines did not correlate with mean normal lung dose at either 4 weeks into treatment or 12 weeks after treatment.

### Association of Plasma Cytokine Concentrations with Likelihood of Severe Respiratory Toxicity

**Figure 5:**
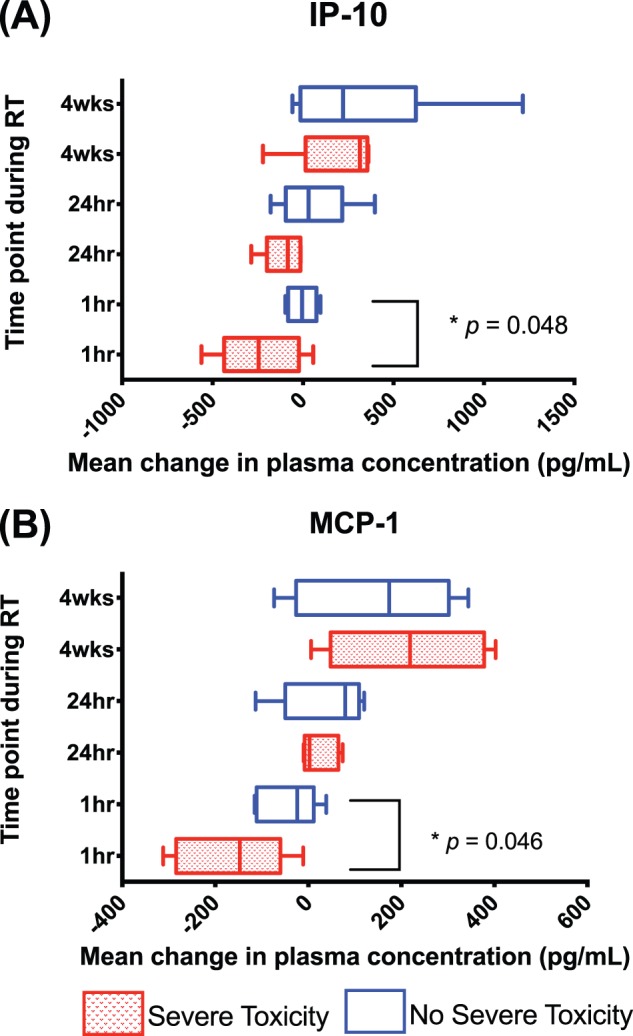
Box and Whisker plot demonstrating median population change of cytokine levels from baseline for IP-10 and MCP-1 during treatment delivery, with 5^th^–95^th^ centiles. Statistically significant differences between patients with severe respiratory toxicities [shaded boxes] and those without [open boxes] are highlighted with associated *p* - values.

**Figure 6:**
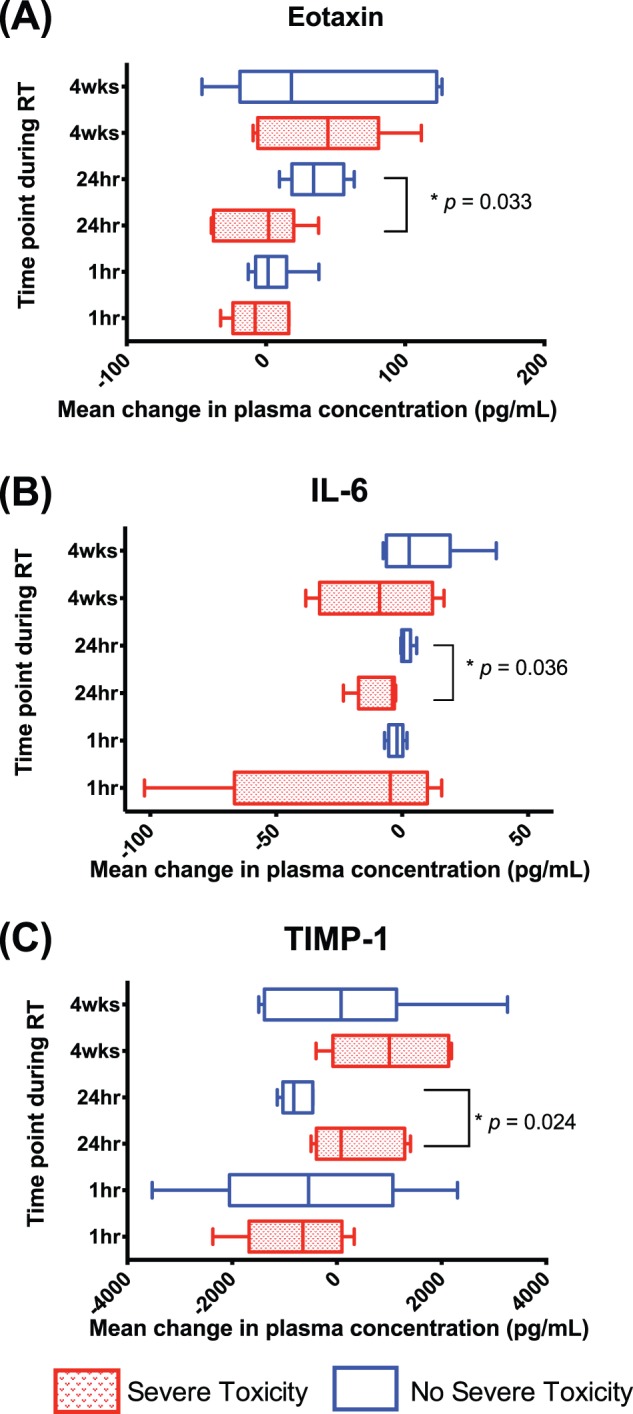
Box and Whisker plot demonstrating median population change of cytokine levels from baseline for Eotaxin, IL-6 and TIMP-1 during treatment delivery, with 5^th^–95^th^ centiles. Statistically significant differences between patients with severe respiratory toxicities [shaded boxes] and those without [open boxes] are highlighted with associated *p* - values.

Five of the twelve patients had severe lung toxicity (CTCAE grade 2 or higher) in this study. Three of these patients were in the chemoRT group and two of these patients received RT alone. Overall, patients with a greater depression in concentrations of MCP-1 and IP-10 levels at 1-hour post the first fraction of radiation subsequently sustained severe lung toxicity (**Figure 5A, 5B** **)**. For those patients with severe toxicities, the mean (+/− standard deviation) reduction of plasma concentration of MCP-1 and IP-10 was 167.0 pg/ml (+/−119.0 pg/ml) and 233.0 pg/ml (+/−232.0 pg/ml), respectively. These levels were significantly more reduced than those in patients who subsequently did not have severe lung toxicity, with corresponding mean reductions of 38.6 pg/ml (+/−62.2 pg/ml), and 4.0 pg/ml (+/−76.7 pg/ml), respectively (*p = *0.05). At 24-hours post the first fraction of radiation, patients with a reduction in concentrations of Eotaxin and IL-6 levels subsequently sustained respiratory toxicity (**Figure 6A, 6B**). For those patients with severe toxicities, the mean (+/− standard deviation) decrease in Eotaxin and IL-6 from pre-treatment levels was 6.8 pg/ml (+/−36.6 pg/ml) and 8.9 pg/ml (+/−8.8 pg/ml), respectively. By comparison, those patients without toxicity had increased Eotaxin and IL-6 levels by a mean (+/− standard deviation) of 31.9 pg/ml (+/−20.4 pg/ml), *p* = 0.03 and 1.4 (+/−2.5 pg/ml) *p* = 0.04, respectively. In contrast, elevated concentrations of TIMP-1 at 24-hours were associated with more severe toxicity (**Figure 6C**). The mean increase (+/− standard deviation) of TIMP-1 in those with severe lung toxicity was 337.0 pg/ml (+/−867.0 pg/ml), versus a decrease of 762.0 pg/ml (+/−292.0 pg/ml) for those without, *p* = 0.02. None of the cytokines tested were significantly different in those with or without severe lung toxicities at 4 weeks into treatment or 12 weeks after completion of treatment.

## Discussion

In this study, we discovered that severe lung toxicity (CTCAE grade 2 or higher) is associated with significant change in some cytokine plasma concentrations from pre-treatment levels in patients receiving definitive RT for NSCLC. Specifically, severe toxicity was associated with the detection of depressed levels of IP-10 and MCP-1 at 1-hour post irradiation, as well as lower levels of Eotaxin and IL-6 at 24 hours post-irradiation as compared to patients who did not subsequently develop severe lung toxicity (**Figure 5** **and** **6** **)**. Levels of TIMP-1 at 24-hours were significantly elevated in patients with severe lung toxicity compared to those without. These early prognostic variations in cytokine levels may represent an individual’s lung sensitivity and subsequent risk for lung toxicity and fibrosis after repetitive exposure to a radiation insult. The detection of a prognostic signal after the first fraction of a 30-fraction course of RT is particular clinical significance, as this may allow for an early intervention during the treatment course. From a clinical perspective, this may manifest as more intensive inter-fractional toxicity assessment and early supportive intervention, potentially with corticosteroids. Alternatively, early prognostic cytokine signature may allow for personalised biological adaptation of the treatment course through increasing or decreasing the intensity of treatment. These cytokine signals were generally less apparent at 4 weeks into therapy. We hypothesize that during a long 6-week course radiation therapy that patients may adjust to the repeated exposures and manifest a less brisk acute inflammatory cytokine response than the initial exposures at the start of therapy. Interpretation of the prognostic significance of cytokines 12 weeks post-therapy is challenging, as this is a complex timepoint due to variability introduced by a broad spectrum of individual patient clinical outcome. At this time some patients will have complete tumoral responses to therapy, whilst others will have stable or progressive disease. In addition, this time may also be influenced by nutritional deficits induced by treatment related esophagitus and dysphagia. The results of this study indicate that early changes in these cytokines should be further investigated as prognostic markers of likelihood of toxicity within patients receiving definitive irradiation for NSCLC.

From a mechanistic perspective, these putative biomarkers of lung injury appear to be reasonable prognostic candidates due to their pro-inflammatory role in various disease states. MCP-1 causes cellular activation of specific functions related to host defence and inflammation, including monocyte, granulocytes and lymphocyte migration [43]. IP-10 also selectively stimulates directional migration of T-cells and monocytes, as well as participating in T cell adhesion [16]. TIMP-1 acts to down-regulate the profibrotic response and is associated with the degree of inflammation in the mucosa of patients with chronic inflammatory states (such as inflammatory bowel disease) [40]. In previous studies, early reduction (within 3–6 hours) of serum levels of IP-10, MCP-1 and TIMP-1 have been previously demonstrated in murine strains more sensitive to fibrosis [C57BL/6] in comparison to more tolerant strains [C3H/HeN] [29], [43], [44]. Later after irradiation, induction of IP-10 and MCP-1 mRNA gene expression up to 6 months post RT in murine models is thought subsequently lead to late tissue fibrosis and subsequent lung damage [16], [45]. IL-6 is a pleiotropic cytokine secreted by T-lymphocytes and involved in maturation of B-lymphocytes, and is thought to mediate clinical fever and regulates inflammation and fibrosis through immune cells [38]. Chen et al. [26] observed early reductions in IL-6 cytokine in patients who sustained pneumonitis, similar to findings in the present study. Eotaxin is a primary mediator of IgE-related allergic inflammatory reactions in lung, which are characteristically associated with an early, transient accumulation of neutrophils and subsequent neutrophil-dependent acute lung inflammatory injury [34]. Plasma levels of Eotaxin have been previously documented in a murine model to initially decrease then subsequently increase over days, similar to the temporal pattern observed in the present study [29].

The concentrations of several cytokines were also demonstrated to be dependent on the dose to normal lung tissue and the irradiated tumour volume (**Figures 2** **and** **3**). In 3D conformal radiotherapy, the dose to the irradiated normal lung tissue (the MLD) is influenced by the number of beams selected, beam angles employed, the location of the tumour, and the degree of sparing of the uninvolved lung. In contrast, the target volume (PTV) is a direct function of the tumour volume and geometric margins applied to account for microscopic disease and delivery error. In this study, both measurements were related to cytokine concentrations at 1-hour post irradiation. Cytokine levels were linearly correlated to the dose to the irradiated normal lung tissue (the MLD), with depression of IP-10, MCP-1 and MCP-3 being the most strongly correlated. Reduction in circulating levels of IL-6, MCP-1 and IP-10 at 1-hour were also correlated with the PTV, although this association was less robust. In particular, this relationship was influenced in particular by the response in one specific patient with a large PTV volume of 1138 cm^3^. Despite this potential confounder, a plausible explanation for a differential relationship between cytokine levels and PTV/MLD is that larger radiation fields (with a larger PTV) which cover mediastinal nodal involvement may not necessarily traverse larger volumes of normal lung parenchyma than smaller tumour volumes located deeper within the lung tissue (which may have a larger MLD). By the same token, this may indicate that IL-6, which significantly correlated with PTV volume but not MLD, may be influenced more by a more generic response in irradiated tissues rather than by response specifically in the bronchoalveolar environment. Conversely, MCP-3, which was correlated to MLD but not PTV volume, may be more specific to a response in pulmonary tissue. IL-6 functions to stimulate the growth and differentiation of B and T lymphocytes and is synthesised by a variety of cells in the lung parenchyma, including alveolar macrophages, lung fibroblasts and type II pneumocytes [38], [44]. The MCP family, including MCP-3 (CCL7), attract cells through activation of their cognate receptor, CC-chemokine receptor (CCR)-2 [32]. A target volume/cytokine response relationship with partial lung irradiation has yet to be described in the context of these cytokines. In addition, a previous study by Arpin et al [25] has previously documented a relationship between serum levels of IL-6 and MLD, however, to our knowledge our findings of a correlation between MLD and IP-10, MCP-1 and MCP-3 are novel discoveries. Further investigation is required to assess whether patients with cytokine responses beyond that of the levels predicted by irradiated target volume or dose to normal lung are more prone to severe toxicity. Again, the ability to detect these dose and volume dependent cytokines at such an early time point during treatment suggests a potential for these biomarkers to be of great clinical utility.

We were able to demonstrate differential plasma cytokine concentrations in patients undergoing RT alone compared to those treated with chemoRT for NSCLC. Whilst previous clinical studies assessing cytokine concentration during lung irradiation have tested for associations between use of chemotherapy and risk of pneumonitis, these have not assessed the differential patterns in plasma cytokine levels for patients receiving RT alone versus chemoRT [24], [25]. In the present study, the overall concentration of cytokines were different dependent upon treatment group for Eotaxin, IL-33, IL-6, MDC, MIP-1α and VEGF. In both groups, the peak elevation in plasma cytokine concentration for MIP-1α occurred at 4 weeks into treatment, whilst peak elevation in Eotaxin and VEGF occurred at 12 weeks after treatment completion. Changes in plasma concentrations of cytokines varied considerably between treatment groups at other time points. Our findings suggest that future studies investigating the kinetics of these plasma levels should not uniformly group patients receiving RT alone and chemoRT together.

In several previous clinical studies of patients treated for NSCLC there have been contradictory reports of associations between RT induced blood cytokine levels and clinical toxicity. A study by Arpin et al. [25] also found changes in IL-6 to be prognostic for radiation pneumonitis, along with combined covariations of IL-6 and IL-10. Similar to our study, TNF-α was not correlated to toxicity. In a study by Crohns et al. [27], VEGF, TNF-α, IL-1β, IL-6 and IL-8 levels in the serum were analysed in patients receiving various regimes of RT with mean (range) dose of 46.9 Gy (30–60 Gy). These investigators were not able to demonstrate any significant changes from the baseline levels of these cytokines (including IL-6) at two weeks or 3 months after the commencement of RT. Similarly, a study by Rűbe et al. [28] measured levels of TGF-β and IL-6 weekly during RT and could not find correlation with symptomatic pneumonitis and plasma level cytokines levels. In their study, patients received a range of treatments including definitive RT alone, definitive chemoRT, low dose twice weekly palliative accelerated RT. This is in contrast to several reports from Anscher’s group [21]–[23], [46] and a study from Zhao et al. [24] indicating that elevation of TGF-β late during RT is associated with risk of pulmonary toxicity. In a study by Chen et al. [26], levels of IL-1α and IL-6 but not TNFα were consistently elevated prior to and throughout treatment in patients whom developed radiation pneumonitis. In this same patient cohort, levels of e-selectin, l-selectin, TGF-β1 and bFGF varied but were not correlated with radiation pneumonitis. Again this cohort had considerable treatment heterogeneity, with 4 of the total 24 patients not having NSCLC, with an average delivered radiation dose of 60–64 Gy, and 3 of 15 patients having had their chemotherapy delivered neoadjuvantly prior to the radiation course. In the context of the previous literature, the strength of our study lies in the standardised treatment characteristics in our patient cohort and the large panel of cytokines tested. We discovered that in addition to IL-6, early changes in plasma levels of four previously unreported cytokines (IP-10, MCP-1, Eotaxin and TIMP-1) were associated with the risk of pulmonary toxicity.

It is important to recognise the limitations of this study. In this study we were not able to control for potential confounding effects of patient stage and volume of irradiated amongst the RT and chemoRT cohorts. Patients treated with RT alone generally had a lower disease stage, smaller PTV and MLD than those patients treated with chemoRT.

### Conclusions

We describe the association between plasma cytokine concentrations and clinical toxicities in a prospective cohort of patients with NSCLC treated with a standardised radiation dose and technique. In our cohort of patients, we observed that inflammatory cytokines were induced in patients with NSCLC both during and after RT. We were able to demonstrate that the concentrations of IL-6, MCP-1, MCP-3 and IP-10 correlated with the mean lung dose. This suggests that these cytokines have potential as *in-vivo* ‘biodosimeters’ of individual radiation dose. In our cohort we also observed the levels of cytokines after RT were different in patients who received concurrent chemotherapy in Eotaxin, IL-33, IL-6, MDC, MIP-1α and VEGF. The change in plasma concentration of IP-10 and MCP-1 at 1-hour post irradiation, and Eotaxin, IL-6 and TIMP-1 at 24-hour post irradiation, were significantly different in those patients who sustained severe lung toxicities. Early decreases in IP-10 and MCP-1 at 1-hour, Eotaxin and IL-6 at 24-hours, and increases in TIMP-1 at 24-hours into RT should be externally validated in other cohorts as potential future biomarkers of severe lung toxicity.

## Supporting Information

Table S1 **Individual patient characteristics and respiratory toxicity.**
(DOCX)

Table S2 **Two-way ANOVA testing the effect of treatment (chemoRT vs RT alone) and sample time point.** All cytokines in which the plasma concentrations varied significantly dependent upon the treatment group are highlighted in bold and by an asterisk (*). The Interaction between treatment group and sample time point is tested, and residual errors are given.
(DOCX)
