# Supplementary material for: A Pattern of Early Radiation-Induced Inflammatory Cytokine Expression Is Associated with Lung Toxicity in Patients with Non-Small Cell Lung Cancer
Source: PLoS One. 2014 Oct 7;9(10):e109560. doi: 10.1371/journal.pone.0109560 (PMC4188745; doi:10.1371/journal.pone.0109560)
Supplement: Table S1 — Individual patient characteristics and respiratory toxicity. (DOCX) [file pone.0109560.s001.docx]

**Supplementary Table 1 – Individual patient characteristics and respiratory toxicity**

| Patient | Treatment | PTV* volume (cc) | Mean Lung Dose (Gy) | CTCAE^θ^ Toxicity ≥ 2 | Stage | Histology |
| --- | --- | --- | --- | --- | --- | --- |
| 1 | RT | 238.14 | 10.94 | N | 2 | Large cell |
| 2 | RT | 87.22 | 7.1 | N | 1 | Adenocarcinoma |
| 3 | RT | 297.3 | 13.38 | Y | 3 | NSCL NOS^£^ |
| 4 | RT | 460.85 | 8.7 | N | 1 | SCC |
| 5 | RT | 140.24 | 5.97 | Y | 1 | SCC |
| 6 | RT | 159.66 | 7.54 | N | 3 | SCC |
|  |  |  |  |  |  |  |
| 7 | ChemoRT | 284.285 | 15.59 | Y | 3 | NSCLC NOS^£^ |
| 8 | ChemoRT | 342.83 | 10.8 | N | 3 | Adenocarcinoma |
| 9 | ChemoRT | 423.7 | 14.4 | N | 3 | SCC |
| 10 | ChemoRT | 1137.66 | 19.14 | Y | 2 | SCC |
| 11 | ChemoRT | 456.594 | 17.16 | Y | 3 | SCC |
| 12 | ChemoRT | 355.27 | 12.46 | N | 2 | Adenocarcinoma |
|  |  | |  |  |  |  |

**PTV (planning target volume),* ^θ^ *CTCAE (Common Terminology Criteria For Adverse Events),* ^£^*NOS (not otherwise specified)*
