# Supplementary material for: A Pattern of Early Radiation-Induced Inflammatory Cytokine Expression Is Associated with Lung Toxicity in Patients with Non-Small Cell Lung Cancer
Source: PLoS One. 2014 Oct 7;9(10):e109560. doi: 10.1371/journal.pone.0109560 (PMC4188745; doi:10.1371/journal.pone.0109560)
Supplement: Table S2 — Two-way ANOVA testing the effect of treatment (chemoRT vs RT alone) and sample time point. All cytokines in which the plasma concentrations varied significantly dependent upon the treatment group are highlighted in bold and by an asterisk (*). The Interaction between treatment group and sample time point is tested, and residual errors are given. (DOCX) [file pone.0109560.s002.docx]

**Supplementary Table 2 – Two-way ANOVA testing the effect of treatment (chemoRT vs RT alone) and sample time point. All cytokines in which the plasma concentrations varied significantly dependent upon the treatment group are highlighted in bold and by an asterisk (*). The Interaction between treatment group and sample time point is tested, and residual errors are given.**

| Cytokine | ANOVA table | Sum of Squares | DF | Mean Square | F ratio (DFn, DFd) | P value |
| --- | --- | --- | --- | --- | --- | --- |
| Eotaxin | Interaction | 1528 | 4 | 382 | F (4, 10) = 1.99 | P = 0.17 |
|  | Treatment Group | 10450 | 1 | 10450 | F (1, 10) = 54.4 | P < 0.01 |
|  | Time Point | 10095 | 4 | 2524 | F (4, 10) = 13.1 | P < 0.01 |
|  | Residuals | 1921 | 10 | 192 |  |  |
|  |  |  |  |  |  |  |
| IL-33 | Interaction | 20530 | 4 | 5132 | F (4, 10) = 0.319 | P = 0.86 |
|  | Treatment Group | 248342 | 1 | 248342 | F (1, 10) = 15.4 | P < 0.01 |
|  | Time Point | 14166 | 4 | 3542 | F (4, 10) = 0.220 | P = 0.92 |
|  | Residuals | 160863 | 10 | 16086 |  |  |
|  |  |  |  |  |  |  |
| IL-6 | Interaction | 1543 | 4 | 386 | F (4, 10) = 7.12 | P < 0.01 |
|  | Treatment Group | 635 | 1 | 635 | F (1, 10) = 11.7 | P < 0.01 |
|  | Time Point | 1122 | 4 | 281 | F (4, 10) = 5.18 | P = 0.02 |
|  | Residual | 542 | 10 | 54.2 |  |  |
|  |  |  |  |  |  |  |
| IP-10 | Interaction | 88303 | 4 | 22076 | F (4, 10) = 27.1 | P < 0.01 |
|  | Treatment Group | 51.6 | 1 | 51.6 | F (1, 10) = 0.0634 | P = 0.81 |
|  | Time Point | 224082 | 4 | 56021 | F (4, 10) = 68.7 | P < 0.01 |
|  | Residuals | 8152 | 10 | 815 |  |  |
|  |  |  |  |  |  |  |
| MCP-1 | Interaction | 48049 | 4 | 12012 | F (4, 10) = 25.2 | P < 0.01 |
|  | Treatment Group | 2186 | 1 | 2186 | F (1, 10) = 4.59 | P = 0.06 |
|  | Time Point | 95884 | 4 | 23971 | F (4, 10) = 50.3 | P < 0.01 |
|  | Residuals | 4765 | 10 | 477 |  |  |
|  |  |  |  |  |  |  |
|  | Interaction | 25.1 | 4 | 6.27 | F (4, 10) = 2.01 | P = 0.17 |
| MCP-3 | Treatment Group | 7.79 | 1 | 7.79 | F (1, 10) = 2.49 | P = 0.15 |
|  | Time Point | 355 | 4 | 88.8 | F (4, 10) = 28.4 | P < 0.01 |
|  | Residuals | 31.3 | 10 | 3.13 |  |  |
|  |  |  |  |  |  |  |
| MDC | Interaction | 760747 | 4 | 190187 | F (4, 10) = 18.0 | P < 0.01 |
|  | Treatment Group | 1.50e+006 | 1 | 1.50e+006 | F (1, 10) = 142 | P < 0.01 |
|  | Time Point | 525298 | 4 | 131325 | F (4, 10) = 12.4 | P < 0.01 |
|  | Residuals | 105930 | 10 | 10593 |  |  |
|  |  |  |  |  |  |  |
| MIP-1α | Interaction | 85391 | 4 | 21348 | F (4, 10) = 12.2 | P < 0.01 |
|  | Treatment Group | 65345 | 1 | 65345 | F (1, 10) = 37.4 | P < 0.01 |
|  | Time Point | 247328 | 4 | 61832 | F (4, 10) = 35.4 | P < 0.01 |
|  | Residuals | 17474 | 10 | 1747 |  |  |
|  |  |  |  |  |  |  |
| MIP-1β | Interaction | 1781 | 4 | 445 | F (4, 10) = 18.1 | P < 0.01 |
|  | Treatment Group | 12.8 | 1 | 12.8 | F (1, 10) = 0.520 | P = 0.49 |
|  | Time Point | 939 | 4 | 235 | F (4, 10) = 9.53 | P < 0.01 |
|  | Residuals | 246 | 10 | 24.6 |  |  |
|  |  |  |  |  |  |  |
| TIMP-1 | Interaction | 1.26e+006 | 4 | 315791 | F (4, 10) = 2.48 | P = 0.11 |
|  | Treatment Group | 224436 | 1 | 224436 | F (1, 10) = 1.76 | P = 0.21 |
|  | Time Point | 3.22e+006 | 4 | 805938 | F (4, 10) = 6.33 | P < 0.01 |
|  | Residuals | 1.27e+006 | 10 | 127330 |  |  |
|  |  |  |  |  |  |  |
| TNF-α | Interaction | 462 | 4 | 115 | F (4, 10) = 1.06 | P = 0.43 |
|  | Treatment Group | 79.5 | 1 | 79.5 | F (1, 10) = 0.729 | P = 0.41 |
|  | Time Point | 380 | 4 | 95.0 | F (4, 10) = 0.872 | P = 0.51 |
|  | Residuals | 1090 | 10 | 109 |  |  |
|  |  |  |  |  |  |  |
| ***VEGF** | Interaction | 540 | 4 | 135 | F (4, 10) = 8.84 | P < 0.01 |
|  | Treatment Group | 931 | 1 | 931 | F (1, 10) = 61.0 | P < 0.01 |
|  | Time Point | 1488 | 4 | 372 | F (4, 10) = 24.4 | P < 0.01 |
|  | Residuals | 153 | 10 | 15.3 |  |  |
